# Plastic Bronchitis in an AIDS Patient with Pulmonary Kaposi Sarcoma

**DOI:** 10.1155/2018/9736516

**Published:** 2018-09-27

**Authors:** Sheila A. Habib, Robert C. Vasko, Jack Badawy, Gregory M. Anstead

**Affiliations:** ^1^Department of Medicine, Division of Pulmonary Diseases and Critical Care Medicine, University of Texas Health at San Antonio (UTH-SA), San Antonio, TX 78229, USA; ^2^Medical Service, Division of Pulmonary Diseases and Critical Care Medicine, South Texas Veterans Health Care System (STVHCS), San Antonio, TX 78229, USA; ^3^Department of Pediatrics, Children's Hospital Los Angeles, Los Angeles, CA 90027, USA; ^4^Department of Medicine, Division of General and Hospital Medicine, UTH-SA, San Antonio, TX 78229, USA; ^5^Department of Medicine, Division of Infectious Diseases, UTH-SA, San Antonio, TX 78229, USA; ^6^Medical Service, Division of Infectious Diseases, STVHCS, San Antonio, TX 78229, USA

## Abstract

Plastic bronchitis is the expectoration of bronchial casts in the mold of the tracheobronchial tree. It is a rare occurrence of unknown etiology that has been primarily described in children with congenital heart disease. In this case report, we present the first reported case of plastic bronchitis in a patient with pulmonary Kaposi sarcoma and underlying HIV infection.

## 1. Introduction

Plastic bronchitis (PB) is the formation of casts in the mold of the tracheobronchial tree, leading to airway obstruction. It has been described in association with a variety of coexisting disorders, but most commonly in children with cyanotic congenital heart disease following the Fontan procedure. To our knowledge, we describe the first case of PB in the setting of human immunodeficiency virus (HIV) infection and pulmonary Kaposi sarcoma (KS).

## 2. Case Presentation

**Figure 1:**
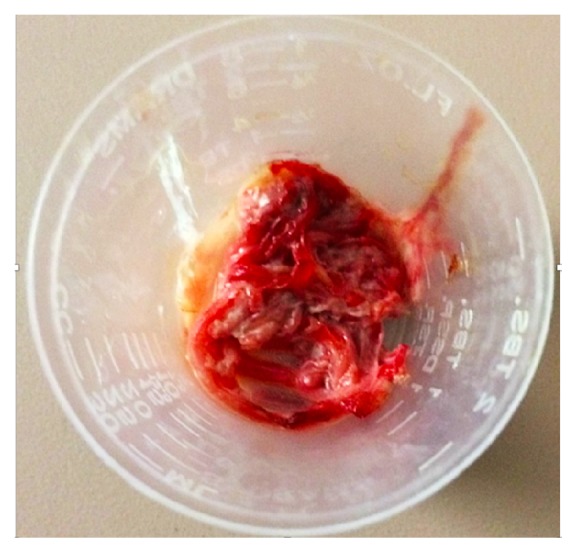
The rubbery red and white material expectorated by the patient.

**Figure 2:**
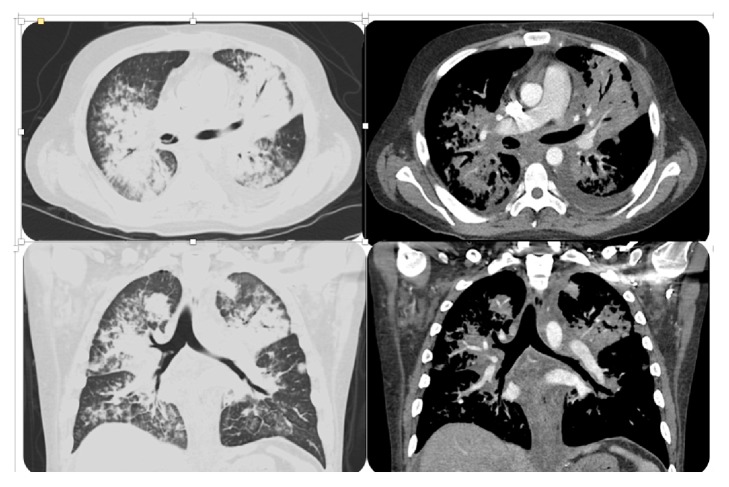
Computed tomography of the chest showed diffuse peribronchovascular consolidative opacities with surrounding ground glass opacities, interlobular septal thickening, and infiltrative soft tissue densities throughout the mediastinum. There is also a linear filling defect in the bronchus intermedius.

**Figure 3:**
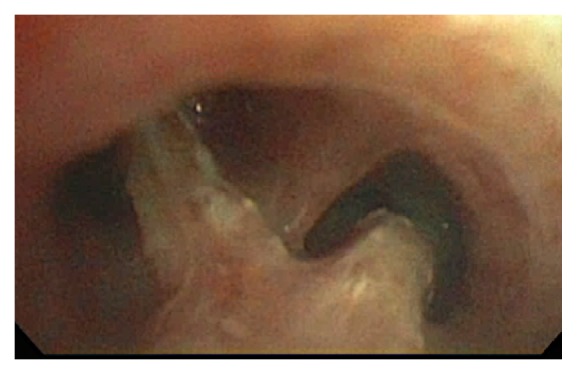
Bronchoscopic image at the level of the main carina with “tissue-like” material evident.

**Figure 4:**
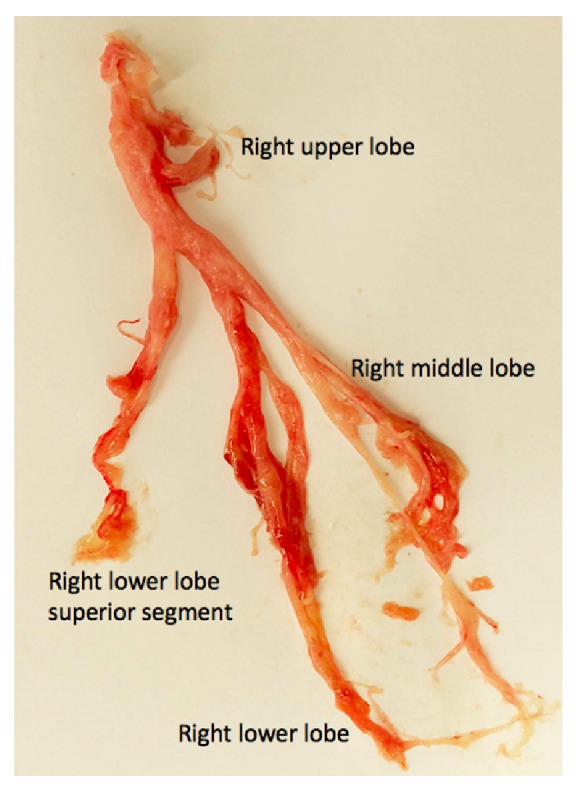
Bronchial cast obtained by flexible bronchoscopy fitting the mold of the right bronchial tree.

**Figure 5:**
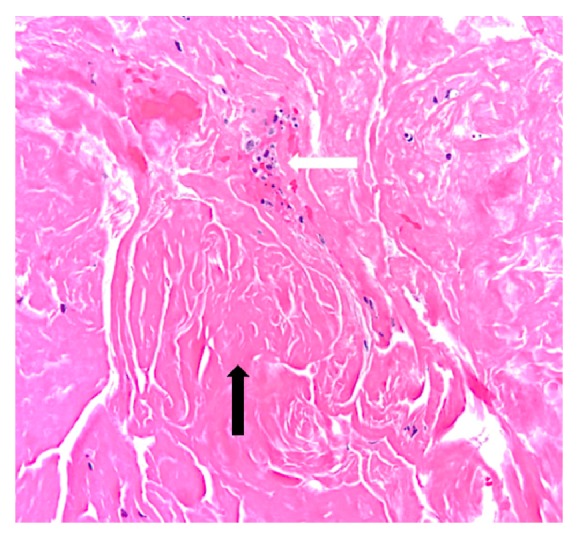
Histopathology of the bronchial cast shows fibrin (black arrow) with few leukocytes (white arrow).

A 25-year-old Hispanic male with HIV infection (CD4 count <40 cells/*μ*L, viral load 307 copies/mL on antiretroviral therapy) and pulmonary KS on chemotherapy presented with progressive dyspnea and cough productive of rubbery red and white material (Figure 1). Physical examination revealed hypoxia, coarse crackles to the bilateral lower lung fields, and multiple violaceous cutaneous plaques. Chest computed tomography showed diffuse peribronchovascular consolidative opacities with surrounding ground glass opacities, interlobular septal thickening, and infiltrative soft tissue densities throughout the mediastinum (Figure 2). Blood and sputum cultures, autoimmune serologic tests, and serologic tests for* Coccidioides* and* Cryptococcus* were negative. Bronchoscopy revealed “tissue-like” material within the tracheobronchial tree, forming casts (Figures 3 and 4). On histopathological analysis, the casts were composed of fibrin with sparse leukocytic infiltrate, consistent with a diagnosis of PB (Figure 5).

Attempts made to clear the fibrinous material from the lung with nebulized dornase alfa, high-frequency oscillation treatments (MetaNeb System (Hill-Rom, Chicago, IL)), and a percussion vest were unsuccessful. Nebulized ipratropium and albuterol and supplemental oxygen by nasal cannula afforded occasional symptomatic relief. Multiple bronchoscopic procedures were performed to remove the fibrinous material from the lung, but it quickly reaccumulated. The expectorated material did not dissolve with tissue plasminogen activator (TPA)* ex vivo*, and thus a trial of nebulized TPA was not conducted. A prednisone taper provided only transient improvement.

In some cases, PB has been due to lymphatic leakage into the bronchi either from surgical trauma or pulmonary lymphatic abnormalities, with resolution of the condition after ligation of the thoracic duct [1]. In this patient, KS of the intrapulmonary lymphatics was likely causing a chyle leakage. Thus, a lymphangiogram was attempted to determine sites of lymphatic leakage that might be amenable to surgical intervention; however, tracer injected into the lymph vessels in the groin area failed to migrate, likely due to lymphatic involvement with KS. Lymphoscintigraphy was also performed, using the hands as injection sites, but no abnormal uptake of tracer within the lungs was demonstrated. Although thoracic duct embolization was offered to the patient, he declined the procedure.

**Figure 6:**
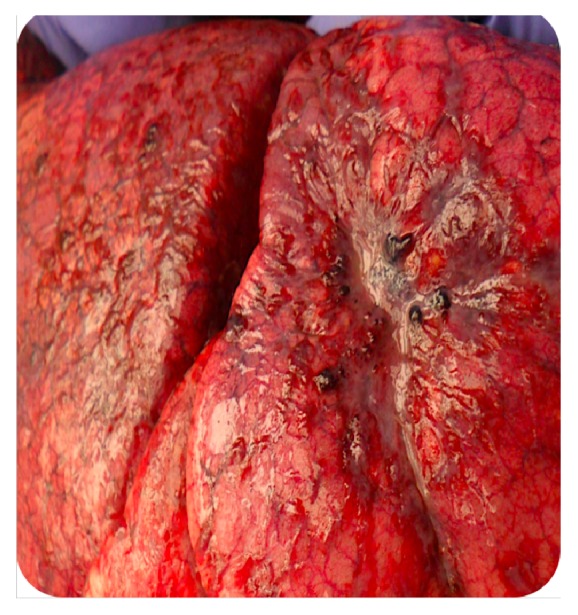
Postmortem finding: hepatization of the lung.

**Figure 7:**
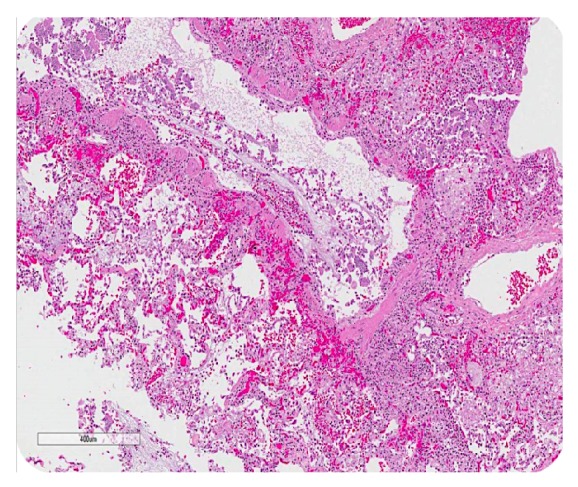
Postmortem finding: longitudinal cross section of a terminal bronchiole filled with mucin and cellular debris.

**Figure 8:**
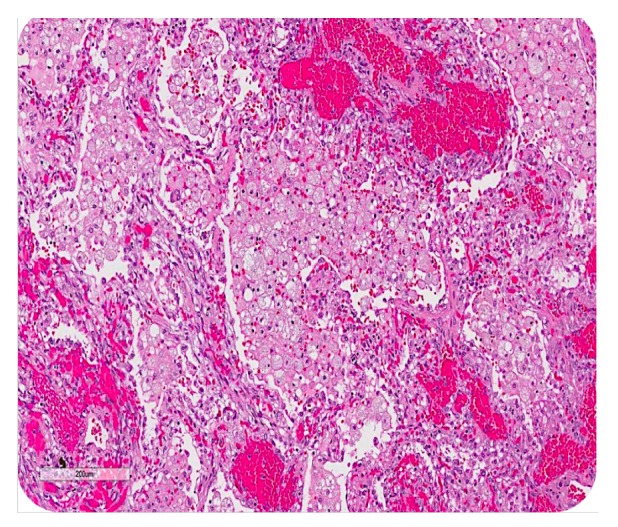
Postmortem finding: mucin-filled alveoli surrounded by angiomatous features of Kaposi sarcoma.

Over approximately three months, the patient was repeatedly readmitted for respiratory distress and ultimately required endotracheal intubation and mechanical ventilation. Repeated bronchoscopy was performed in an effort to clear the casts, but it was unsuccessful. While on the ventilator, he empirically received multiple therapies for the reduction of lymphatic flow (including total parenteral nutrition (TPN), midodrine, and octreotide) and the treatment of KS (with sirolimus) to curb cast production. Unfortunately, the patient developed refractory respiratory failure and was transitioned to comfort measures. An autopsy revealed extensive pulmonary KS with hepatization of the lung and near obliteration of the normal alveolar architecture with copious mucin and cellular debris within the airways (Figures 6, 7, and 8).

## 3. Discussion

Plastic bronchitis is a rare condition of unclear pathogenesis characterized by the expectoration of casts in the mold of the tracheobronchial tree. It was first described by Galen in the second century AD as* venae arteriosae expectorantii*, which translates literally to “expectorated arteries and veins” [1, 2]. Since that time, PB has been described in association with bronchial inflammation (due to asthma [3–5], allergic bronchopulmonary aspergillosis (ABPA) [6], cystic fibrosis [7], influenza [8], and pneumonia); cardiac anomalies (especially following the palliative Fontan procedure [9]); and disorders of lymphatic drainage (lymphangiectasia and lymphangiomatosis [10–13]). Patients with PB typically present with nonspecific symptoms of cough, wheezing, dyspnea, and hypoxemia [1, 14]. In a minority of patients,* ventilgeraeusch* (“sound of a fan”) or* bruit de drapeau* (“sound of a flag snapping”) may be present and indicate subtotal airway obstruction [1]. Diagnosis is made by visualization of casts, either in expectorated material or via bronchoscopy. Casts typically appear as a white branching mold of the tracheobronchial tree. In our case, we suspect the white-red coloration to be secondary to the vascular nature of KS and the propensity for bleeding. Multiple classification schemes have been proposed for PB [2, 15]. Seear et al. reviewed nine cases with bronchial cast formation and characterized two distinct groups. Type 1 (or inflammatory) casts are composed of fibrin and have a dense eosinophilic infiltrate. They are typically observed in the setting of underlying bronchial disease (such as asthma, cystic fibrosis, and ABPA). Type 2 (or acellular) casts consist mainly of mucin with little cellular infiltrate. These are most commonly observed following the Fontan procedure but may also be seen in noninflammatory causes of PB [15, 16]. Therapeutic approaches to PB are largely anecdotal and focus on (1) facilitating the removal or expectoration of casts and (2) treatment of the underlying etiology. Cast removal may be achieved mechanically (by bronchoscopy [17, 18] and chest physiotherapy) and/or by pharmacologic therapies. PB with type 1/inflammatory casts may respond to anti-inflammatory therapies such as oral and inhaled corticosteroids, bronchodilators, mucolytics [19, 20], and macrolide antibiotics [21]. On the other hand, those with type 2/acellular casts may benefit from optimization of hemodynamics, aerosolized fibrinolytics [14, 16, 19, 22], or thoracic duct ligation [1]. In both cases, aggressive treatment of the underlying etiology provides the most durable relief.

The development of PB has not been previously described in patients with KS, an angioproliferative disorder of the vascular and lymphatic endothelium. KS is well known to cause lymphatic obstruction and lymphedema of the extremities but lymphatic stasis in the lungs has not been well described [23, 24]. Other disorders of the pulmonary lymphatic system (such as lymphangiectasia and lymphangiomatosis) have been associated with the development of PB [11, 12]. Treatment of these disorders focuses on decreasing chyle production by dietary modifications (i.e., a low fat diet excluding long-chain triglycerides, TPN [25]), pharmacologic therapies (including octreotide [26], midodrine [27]), and/or surgical intervention (i.e., thoracic duct ligation) [1]. In this case, the patient empirically received TPN and trials of pharmacologic therapies; however, he continued to produce bronchial casts with frequent airway obstruction. Thoracic duct ligation was considered but was ultimately deferred due to critical illness. Despite empiric aggressive treatment, our patient had persistent bronchial obstruction due to ongoing cast formation and developed refractory respiratory failure. Postmortem analysis revealed extensive pulmonary KS and loss of alveolar architecture that ultimately resulted in refractory respiratory failure.

Plastic bronchitis, although rare, is a life-threatening condition that has been seen in multiple underlying pulmonary conditions. This case report highlights an unusual cause of PB and the need for further investigation into its pathogenesis and therapeutic measures.

## References

[1] Eberlein M. H., Drummond M. B., Haponik E. F. (2008). Plastic bronchitis: A management challenge. *The American Journal of the Medical Sciences*.

[2] Madsen P., Shah S. A., Rubin B. K. (2005). Plastic bronchitis: New insights and a classification scheme. *Paediatric Respiratory Reviews*.

[3] Pawar S. S., Chun R. H., Rao A. R., Kerschner J. E. (2011). Management of plastic bronchitis in a child with mild intermittent asthma. *Annals of Otology, Rhinology & Laryngology*.

[4] Tonan M., Sawada M., Tsuchiya K. (2012). Successful treatment of severe asthma-associated plastic bronchitis with extracorporeal membrane oxygenation. *Journal of Anesthesia & Clinical Research*.

[5] Kim E. J., Park J. E., Kim D. H., Lee J. (2012). Plastic Bronchitis in an Adult with Asthma. *Tuberculosis and Respiratory Diseases*.

[6] Sanerkin N. G., Seal R. M., Leopold J. G. (1966). Plastic bronchitis, mucoid impaction of the bronchi and allergic broncho-pulmonary aspergillosis, and their relationship to bronchial asthma.. *Annals of Allergy, Asthma & Immunology*.

[7] Mateos-Corral D., Cutz E., Solomon M., Ratjen F. (2009). Plastic bronchitis as an unusual cause of mucus plugging in cystic fibrosis. *Pediatric Pulmonology*.

[8] Zhang J., Kang X. (2015). Plastic bronchitis associated with influenza virus infection in children: A report on 14 cases. *International Journal of Pediatric Otorhinolaryngology*.

[9] Larue M., Gossett J. G., Stewart R. D., Backer C. L., Mavroudis C., Jacobs M. L. (2012). Plastic Bronchitis in Patients With Fontan Physiology: Review of the Literature and Preliminary Experience With Fontan Conversion and Cardiac Transplantation. *World Journal for Pediatric and Congenital Heart Surgery*.

[10] Stoddart A., Dincer H. E., Iber C., Tomic R., Bhargava M. (2014). Chyloptysis causing plastic bronchitis. *Respiratory Medicine Case Reports*.

[11] Wiggins J., Sheffield E., Jeffery P. K., Geddes D. M., Corrin B. (1989). Bronchial casts associated with hilar lymphatic and pulmonary lymphoid abnormalities. *Thorax*.

[12] Nair L. G., Kurtz C. P. (1996). Lymphangiomatosis presenting with bronchial cast formation. *Thorax*.

[13] Languepin J., Scheinmann P., Mahut B. (1999). Bronchial casts in children with cardiopathies: The role of pulmonary lymphatic abnormalities. *Pediatric Pulmonology*.

[14] Avitabile C. M., Goldberg D. J., Dodds K., Dori Y., Ravishankar C., Rychik J. (2014). A multifaceted approach to the management of plastic bronchitis after cavopulmonary palliation. *The Annals of Thoracic Surgery*.

[15] Seear M., Hui H., Magee F., Bohn D., Cutz E. (1997). Bronchial casts in children: A proposed classification based on nine cases and a review of the literature. *American Journal of Respiratory and Critical Care Medicine*.

[16] Rubin B. K. (2016). Plastic Bronchitis. *Clinics in Chest Medicine*.

[17] Sriratanaviriyakul N., Lam F., Morrissey B. M., Stollenwerk N., Schivo M., Yoneda K. Y. (2015). Safety and Clinical Utility of Flexible Bronchoscopic Cryoextraction in Patients with Non-neoplasm Tracheobronchial Obstruction. *Journal of Bronchology & Interventional Pulmonology*.

[18] Ishman S., Book D. T., Conley S. F., Kerschner J. E. (2003). Plastic bronchitis: An unusual bronchoscopic challenge associated with congenital heart disease repair. *International Journal of Pediatric Otorhinolaryngology*.

[19] Gibb E., Blount R., Lewis N. (2012). Management of plastic bronchitis with topical tissue-type plasminogen activator. *Pediatrics*.

[20] Lis G., Cichocka-Jarosz E., Jedynak-Wasowicz U., Glowacka E. (2014). Add-on treatment with nebulized hypertonic saline in a child with plastic bronchitis after the Glenn procedure. *Jornal Brasileiro de Pneumologia*.

[21] Schultz K. D., Oermann C. M. (2003). Treatment of cast bronchitis with low-dose oral azithromycin. *Pediatric Pulmonology*.

[22] Heath L., Ling S., Racz J. (2011). Prospective, longitudinal study of plastic bronchitis cast pathology and responsiveness to tissue plasminogen activator. *Pediatric Cardiology*.

[23] Gasparetto T. D., Marchiori E., Lourenço S. (2009). Pulmonary involvement in Kaposi sarcoma: Correlation between imaging and pathology. *Orphanet Journal of Rare Diseases*.

[24] Ramdial P. K., Chetty R., Singh B., Singh R., Aboobaker J. (2006). Lymphedematous HIV-associated Kaposi's sarcoma. *Journal of Cutaneous Pathology*.

[25] Sriram K., Meguid R. A., Meguid M. M. (2016). Nutritional support in adults with chyle leaks. *Nutrition Journal *.

[26] Helin R. D., Angeles S. T. V., Bhat R. (2006). Octreotide therapy for chylothorax in infants and children: A brief review. *Pediatric Critical Care Medicine*.

[27] Liou D. Z., Warren H., Maher D. P. (2013). A novel therapeutic for refractory chylothorax. *CHEST*.

